# Inhibitory Effects of Trifluoperazine on Peripheral Proinflammatory Cytokine Expression and Hypothalamic Microglia Activation in Obese Mice Induced by Chronic Feeding With High-Fat-Diet

**DOI:** 10.3389/fncel.2021.752771

**Published:** 2021-10-26

**Authors:** Hui-Ting Huang, Pei-Chun Chen, Po-See Chen, Wen-Tai Chiu, Yu-Min Kuo, Shun-Fen Tzeng

**Affiliations:** ^1^Institute of Life Sciences, College of Bioscience and Biotechnology, National Cheng Kung University, Tainan, Taiwan; ^2^Institute of Physiology, College of Medicine, National Cheng Kung University, Tainan, Taiwan; ^3^Department of Psychiatry, College of Medicine, National Cheng Kung University, Tainan, Taiwan; ^4^Department of Biomedical Engineering, College of Engineering, National Cheng Kung University, Tainan, Taiwan; ^5^Institute of Basic Medical Sciences, Department of Cell Biology and Anatomy, College of Medicine, National Cheng Kung University, Tainan, Taiwan

**Keywords:** inflammation, cytokine, microglia, gliosis, dopamine type 2 receptor

## Abstract

Microglia and astrocytes are the glial cells of the central nervous system (CNS) to support neurodevelopment and neuronal function. Yet, their activation in association with CNS inflammation is involved in the initiation and progression of neurological disorders. Mild inflammation in the periphery and glial activation called as gliosis in the hypothalamic region, arcuate nucleus (ARC), are generally observed in obese individuals and animal models. Thus, reduction in peripheral and central inflammation is considered as a strategy to lessen the abnormality of obesity-associated metabolic indices. In this study, we reported that acute peripheral challenge by inflammagen lipopolysaccharide (LPS) upregulated the expression of hypothalamic dopamine type 2 receptor (D2R) mRNA, and chronic feeding by high-fat-diet (HFD) significantly caused increased levels of D2R in the ARC. The *in vitro* and *in vivo* studies indicated that an FDA-approved antipsychotic drug named trifluoperazine (TFP), a D2R inhibitor was able to suppress LPS-stimulated activation of microglia and effectively inhibited LPS-induced peripheral inflammation, as well as hypothalamic inflammation. Further findings showed daily peripheral administration intraperitoneally (i.p.) by TFP for 4 weeks was able to reduce the levels of plasma tumor necrosis factor-α (TNF-α) and interleukin-1β (IL-1β) in accompany with lower levels of plasma glucose and insulin in obese mice receiving HFD for 16 weeks when compared those in obese mice without TFP treatment. In parallel, the activation of microglia and astrocytes in the ARC was also inhibited by peripheral administration by TFP. According to our results, TFP has the ability to suppress HFD-induced ARC gliosis and inflammation in the hypothalamus.

## Introduction

Obesity caused by excessive dietary intake is not only a causal factor in the development of cardiovascular disease but also a harmful stimulus to induce neurodegeneration in the central nervous system (CNS) and cognitive dysfunction (Miller and Spencer, 2014). The hypothalamus is critical for regulating food intake and energy expenditure (Konner et al., 2009). The hypothalamus consists of two centers, including a satiety center and a feeding center, which reciprocally coordinate to maintain a set point for body weight control. Hypothalamic paraventricular nuclei and arcuate nuclei (ARC) play the role in neuroendocrine regulation and secrete a hormone-releasing hormone to act on the anterior pituitary gland for hormone release (Sohn et al., 2013).

Chronic systemic low-grade inflammation is a hallmark of obesity (Hotamisligil, 2006; Kanneganti and Dixit, 2012). The proinflammatory cytokines (i.e., TNF-α, IL-1β, IL-6) and inflammatory markers (such as CRP, TLRs) have been detected in the plasma of obese individuals and fat-fed animals. Notably, obesity-associated brain inflammation occurs significantly in the hypothalamic ARC region (Purkayastha and Cai, 2013; Miller and Spencer, 2014; Pimentel et al., 2014; Lee et al., 2020). In a rodent study, hypothalamic inflammation occurred at the onset of high-fat-diet (HFD) feeding (Thaler et al., 2012; Baufeld et al., 2016). Gliosis, a reactive change in astrocytes (CNS supporting cell population) and microglia (CNS resident macrophages), also occurs in the hypothalamus of obese rodents and humans (Thaler et al., 2012; Baufeld et al., 2016; Cai and Khor, 2019), which is considered as the cellular contributors of obesity-associated hypothalamic inflammation (Lee et al., 2020). Since ARC is adjacent to the median eminence, a circumventricular organ that lacks the effective blood-brain barrier (Williams, 2012; Miller and Spencer, 2014), HFD-induced peripheral inflammatory regulators or free fatty acids are more easily permeable into this region, activate ARC glial cells, and initiate hypothalamic inflammation(Guillemot-Legris and Muccioli, 2017; Cai and Khor, 2019). Particularly, ARC microglia, rapidly respond to acute HFD feeding and are sustainably reactive during chronic HFD feeding (Thaler et al., 2012; Valdearcos et al., 2014; Lee et al., 2020). The activation of ARC microglia and astrocytes has been suggested to be involved in HFD-induced leptin and insulin resistance and disturbance of energy homeostasis (Chowen et al., 2013). Examination of cytokine expression in HFD-fed rats has shown that proinflammatory cytokines (IL-6 and TNF-α) were increased in the hypothalamus within 1–3 days and extending to 3 weeks after HFD feeding (Thaler et al., 2012; Jastroch et al., 2014). Similar observations have reported that IL-1β expression was upregulated in the hypothalamus 3 days after the initiation of HFD feeding in mice (Baufeld et al., 2016). However, the expression of these proinflammatory cytokines in rats and mice declines in the weeks after HFD feeding (Baufeld et al., 2016). Nevertheless, prolonged microglial activation and demyelination were observed in the ARC of mice treated with chronic HFD feeding for months (Huang et al., 2019). Thus, the inhibition of hypothalamic inflammation is considered an effective strategy for the control of glucose homeostasis (Milanski et al., 2009; Miller and Spencer, 2014).

Trifluoperazine (TFP) is a prescribed antipsychotic drug that has been used to suppress psychotic activity by inhibiting the postsynaptic dopamine type 2 receptor (D2R; Marques et al., 2004). The compound given by a single injection into the animals not only has an immunosuppressive ability to inhibit cytokine production and release from immune cells (Roudebush et al., 1991; Park et al., 2019) but also shows anti-cancer effects through different signaling pathways (Xia et al., 2019). Thus, the aim of the study was to determine whether TFP has a potential effect on the attenuation of HFD-induced inflammation. Although TFP did not attenuate the body weight of obese mice induced by chronic HFD feeding, the *in vivo* findings in conjunction with the *in vitro* observation that exposure to TFP significantly reduced LPS-induced proinflammatory cytokine expression in primary microglia demonstrate that treatment with TFP effectively attenuated HFD-induced elevation in blood glucose, systemic inflammation, and ARC gliosis.

## Materials and Methods

### Cell Culture

Primary microglia were collected from mixed glial cultures that were prepared *via* homogenization of cortical tissues dissected from mouse and rat pups at postnatal day (PD) 1–2 (Wang et al., 2016). Alternatively, hypothalamic mixed glial cells were prepared from mouse pups at PD1–2. The tissues were filtered through a 70-μm nylon filter mesh, and after centrifugation, the cell pellet was re-suspended in DMEM/F-12 medium (Thermo Fisher Scientific) containing 10% fetal bovine serum (Gibco). The cells were seeded onto poly-D-lysine (PDL; Sigma-Aldrich)-coated T-75 culture flasks and incubated in 5% CO_2_ at 37°C for 7–8 days. After microglia were collected using the shake-off method, these cells were re-plated onto coverslips in 24-well plates at a density of 5 × 10^4^ cells/well or in 60-mm Petri dishes at a density of 1 × 10^6^ cells/dish in DMEM/F-12 medium containing 10% FBS for 3 h. The cultures were treated with 1 μM TFP (Sigma-Aldrich) in the presence of LPS (10 ng/ml; Sigma-Aldrich) in DMEM containing N1 serum supplement (Thermo Fisher Scientific) for 3 h to examine isolectin B4 (IB4) and immunofluorescence for inducible nitric oxide synthase (iNOS) or for 6 h to investigate TNF-α and IL-1β mRNA expression. Alternatively, mouse mixed glia was prepared from the hypothalamus of pups at the age of PD 1–2 and re-plated onto PDL-coated coverslips in a 24-well plate at a density of 5 × 10^4^ cells per well. After incubation in 5% CO_2_ at 37°C for 5 days, the cultures were treated in DMEM/F-12 medium without or with 1 μM TFP in the presence of LPS (10 ng/ml) for 3 h. The cultured media were collected for the measurement of TNF-α and IL-1β levels. Animal use for primary microglial preparation was approved by the National Cheng Kung University Institutional Animal Care and Use Committee, Tainan, Taiwan (IACUC approval number: 106060). The methods were performed in accordance with relevant guidelines and regulations.

### Animals

Eight-week-old male C57BL/6 mice were purchased from National Cheng Kung University Laboratory Animal Center^1^, and randomly paired-housed in cages with free access to food and water *ad libitum* under standard room conditions (room temperature: 23 ± 2°C; humidity: 58 ± 2%; 12-h light/dark cycle). To induce obesity in mice, the animals were fed an HFD containing 61.6% kcal from fats, 18.1% from proteins, and 20.3% from carbohydrates (Rodent Purified Diet #58Y1; TestDiet, St. Louis, MO, USA). The animals receiving a normal diet (Laboratory Rodent Diet #5001; LabDiet, St. Louis, MO, USA) were referred to as the chow group. All the experiments were performed in compliance with ARRIVE (Animal Research: Reporting *in vivo* Experiments) Guidelines^2^ and strictly following the 3Rs (Replacement, Reduction, and Refinement) to avoid unnecessary sacrifice. Animal experiments through the performance of the following methods were approved by the National Cheng Kung University Institutional Animal Care and Use Committee, Tainan, Taiwan (IACUC approval number: 106060). The animals were anesthetized and sacrificed by i.p. injection with Zoletil 50 (Virbac Taiwan Co., Ltd.; 5× dilution in saline, 0.05–0.06 ml/10 g). The choice of anesthetics was recommended by the veterinary specialist at the university animal center to effectively reduce pain in animals. The experimental protocol is outlined in Supplementary Figure 1.

### Peripheral Injection of LPS and TFP

A bolus of injection of LPS (1 mg/kg) combined with saline or TFP (2 mg/kg) by an i.p. route was performed in the afternoon on 8-week-old C57BL/6 mice. Animals that received saline served as the control group (vehicle). Blood samples were collected at 3 h from the retro-orbital sinuses of anesthetized mice using a heparinized capillary tube (Tsai et al., 2018). Alternatively, under anesthesia, blood samples were collected at 6 h *via* cardiac puncture using a 26G needle rinsed with 10 μl of heparin (5,000 IU/ml; Leo Pharmaceutical, Ltd., Denmark). Animals were then sacrificed, and hypothalamic tissues were removed for measurement of TNF-α and IL-β mRNA levels.

TFP (2 mg/kg/injection) administration through the i.p. route began at 12 weeks post HFD feeding. A single afternoon injection by TFP was performed daily and continued for 4 weeks. Based on research studies in mice (Park et al., 2019; Sylvain et al., 2021), the low dosage of TFP was chosen to avoid side effects from the 4-week injection. The animal group includes chow-vehicle, HFD-vehicle, chow-TFP, and HFD-TFP. Blood samples and brain tissues were collected after animals were anesthetized.

### Measurement of Cytokines

The mouse hypothalamic mixed glial culture media or the blood samples from mice were collected at the different experimental time points as described above. After centrifugation, the supernatant was used to measure TNF-α and IL-1β using a murine TNF-α Quantikine ELISA Kit and murine IL-1β/IL-1F2 Quantikine ELISA Kit following the protocol provided by the vendor (R&D system; Table 1).

**Table 1:** List of reagents (antibodies and assay kits) in the study.

Reagents	Manufacturer (RRID)
Monoclonal rat anti-CD11b antibody	BD Biosciences, Cat#550282 RRID:AB_393577
Polyclonal rabbit anti-D2R	Biorbyt, Cat# orb10515, RRID:AB_10747533
Polyclonal rabbit anti-GFAP antibody	Millipore, RRID:AB_2109645
Polyclonal rabbit anti-Iba1 antibody	Wako, RRID:AB_839504
Polyclonal rabbit anti-iNOS antibody	Calbiochem, RRID: AB_212220
Biotin-conjugated isolectin B4 (IB4)	Sigma-Aldrich, Cat#L2140
Biotinylated anti-rabbit IgG	Abcam, RRID: AB_954902
Cy3-conjugated Streptavidin	Thermo Fischer, Cat#434315
Alexa Fluo 488- Streptavidin	Thermo Fisher, RRID: AB_2336881
Mouse TNF-α Quantikine ELISA Kit	R&D Systems, RRID:AB_2877064
Mouse IL-1β/IL-1F2 Quantikine ELISA Kit	R&D Systems, Cat# MLB00C
Mercodia Mouse Insulin ELISA Kit	Mercodia, RRID:AB_2783837
HDL and LDL/VLDL Quantification Colorimetric/Fluorometric Kit	BioVision, Cat# K613
Triglyceride Quantification Colorimetric/Fluorometric Kit	BioVision, Cat# K622

### Assays for Metabolic Indices

The blood samples used for the measurement of glucose were collected using a heparinized capillary tube from animals after fasting for 15 h. Roche Accu-Chek^®^ blood glucose meters were used to measure the amount of blood glucose in the animal groups. In addition, the blood samples collected from the fasting animals were centrifuged, and the supernatant was used to measure plasma insulin (Table 1) and other metabolic markers, including high-density lipoprotein (HDL), low-density lipoprotein/very-low-density lipoprotein (LDL/VLDL), and triglyceride, using Quantification Colorimetric/Fluorometric Kits (Table 1).

### Collection of Adipose Tissues

After perfusion, white adipose tissue (WAT) was collected from epididymal adipose tissue, and brown adipose tissue (BAT) was collected from interscapular brown adipose tissue for weight measurement on an electronic analytical balance (ATX224; SHIMADZU, Japan).

### Quantitative Real-Time Polymerase Chain Reaction (QPCR)

Animals were anesthetized with Zoletil 50 (Virbac Taiwan Co., Ltd.; 5X dilution in saline, 0.05–0.06 ml/10 gm) by i.p. injection and then perfused with 0.9% saline in diethylpyrocarbonate (DEPC; Sigma)-treated distilled water. Hypothalamic tissues were removed and homogenized in TRIzol^TM^ (Invitrogen) for RNA extraction. cDNA generation using MMLV reverse transcriptase (Invitrogen) and PCR amplification by Fast SYBR^®^ Green Master Mix (Applied Biosystems) were previously described (Huang et al., 2020). We used Primer-BLAST software provided by the National Center for Biotechnology Information to design primers that were manufactured by Taiwan Genomics. The level of cyclophilin A (CyPA) was used as an internal control. StepOne Software v2.1 (Applied Biosystems) was used to determine the cycle threshold fluorescence values. The expression level of the target genes relative to the internal control is presented as 2−ΔCT, where ΔCT = (Ct target gene-Ct CyPA). The sequences of the specific primers for TNF-α, IL-1β, D2R, and CyPA are as follows: rat TNF-α (NM_012675): Forward 5’-CATCCGTTCTCTACCCAGCC-3’,Reverse 5’-AATTCTGAGCCCGGAGTTGG-3’; rat IL-1β (NM_031512.2): Forward 5’-CCTATGTCTTGCCCGTGGAG-3’ Reverse 5’-CACACACTAGCAGGTCGTCA-3’; rat CyPA (NM_017101.1): Forward 5’-CGTCTGCTTCGAGCTGTTTG-3’, Reverse 5’-GTAAAATGCCCGCAAGTCAA-3’; murine TNF-α (NM_008361.4) Forward 5’-CCGGACTCCGCAAAGTCTAA-3’, Reverse 5’-ACCGTCAGCCGATTTGCTAT-3’; murine IL-1β (NM_008361.4): Forward 5’-TGCCACCTTTTGACAGTGATGA-3’, Reverse 5’-AAGGTCCACGGGAAAGACAC-3’; murine D2R (NM_010077.3): Forward 5’-CCATTGTCTGGGTCCTGTCC-3’, Reverse 5’-CTGCTACGCTTGGTGTTGAC-3’; and murine CyPA (NM_008907.1): Forward 5’-CGTCTGCTTCGAGCTGTTTG-3’, Reverse 5’-GTAAAATGCCCGCAAGTCAA-3’.

### Double Immunofluorescence

The cell cultures were fixed in PBS containing 4% paraformaldehyde for 10 min and then treated with 0.1% Triton-X-100 in PBS at room temperature for 30 min. Rat microglia were incubated with rabbit anti-iNOS antibody (1:400) and biotin-conjugated IB4 (1:200) in PBS containing 5% horse serum overnight at 4°C, followed by biotinylated anti-rabbit IgG (1:200) for anti-iNOS antibody and Alexa Fluor 488 (1:200) for IB4 for 1 h at room temperature. The cultures were incubated with PBS containing Cy3–avidin (1:200) for another 45 min. Alternatively, mouse mixed glial cells were incubated with anti-GFAP antibody (1:400) and biotin-conjugated IB4 (1:200) overnight at 4°C. The cells were reacted with biotinylated anti-rabbit IgG (1:200) and Alexa Fluor 488 (1:200) at room temperature for 1 h and then Cy3-avidin (1:200) for another 45 min. The immunostained cells were visualized and photographed under a Nikon E800 fluorescence microscope equipped with a CCD camera. The antibodies are listed in Table 1.

### Brain Tissue Immunostaining

The brain samples removed from animals were fixed overnight in 4% paraformaldehyde and then transferred to tubes containing 30% (w/v) sucrose in PBS until the tissues sank to the bottom of the tube. The tissues were mounted in Tissue Tek optimal cutting temperature compound (Electron Microscopy Sciences, Torrance, CA, USA) and then sectioned at 20-μm thickness. The floating brain sections were treated with 1% Triton-X-100 in PBS at 4°C overnight, followed by incubation with primary antibodies in PBS containing 0.1% Triton X-100 and 1% horse serum at 4°C overnight. The tissue sections were then incubated with biotinylated secondary antibodies for 1 h and with Alexa Fluor 488 or Cy3–avidin (1:200) for another 45 min. The immunostained tissues were observed under an Olympus FLUOVIEW FV1000 confocal laser scanning microscope (FV1000, Japan) at wavelengths of 405, 488, or 594 nm. The primary antibodies used in the study are listed in Table 1. Alternatively, brain sections were permeabilized in 0.3% Triton-X-100 in PBS for 30 min, incubated with rabbit anti-D2R antibody (Table 1) in PBS at 4°C overnight, and treated with biotinylated anti-rabbit IgG (1:200) in PBS for 1 h. The brain sections were then reacted with Vectastain ABC reagent (Vector Labs; Cat# PK-6100) containing 3,3’-diaminobenzidine and nuclear counterstaining by hematoxylin. The immunostained sections were visualized and photographed under a Nikon E800 microscope equipped with a CCD camera.

### Determination of Microglial Activation and Astrocytic Hypertrophy

The measurement of microglial and astrocytic activation was performed as previously described (Huang et al., 2019, 2020). The cell number and cell body size of Iba1^+^ microglia and the intensity of GFAP immunoreactivity in the ARC were analyzed using National Institutes of Health (NIH) ImageJ analysis software (Rueden et al., 2017). In general, randomly selected images per brain section were merged and captured in multiple 1-μm-thick steps using an Olympus FLUOVIEW FV1000 confocal laser scanning microscope. Images that were used for quantification were captured from 7–9 brain sections for Iba1 immunostaining and six brain sections for GFAP immunostaining from three animals per group. The microglial cell numbers are shown as the number of cells in an image with a size of 0.1 mm^2^. The GFAP intensity data were normalized to those of the chow group (100%).

### Statistical Analysis

The presence of significant differences between each group (chow-vehicle, chow-TFP, HFD-vehicle, HFD-TFP) at different time points observed in this study was determined using two-way ANOVA with Sidak’s multiple comparisons for the *in vivo* study or two-tailed Student’s *t*-test for *in vitro* experiments. Data are presented as the mean ± SEM. The statistical significance was set as *P* < 0.05.

## Results

### Trifluoperazine Attenuates the Production of Proinflammatory Mediators Induced by Inflammation Challenge

**Figure 1:**
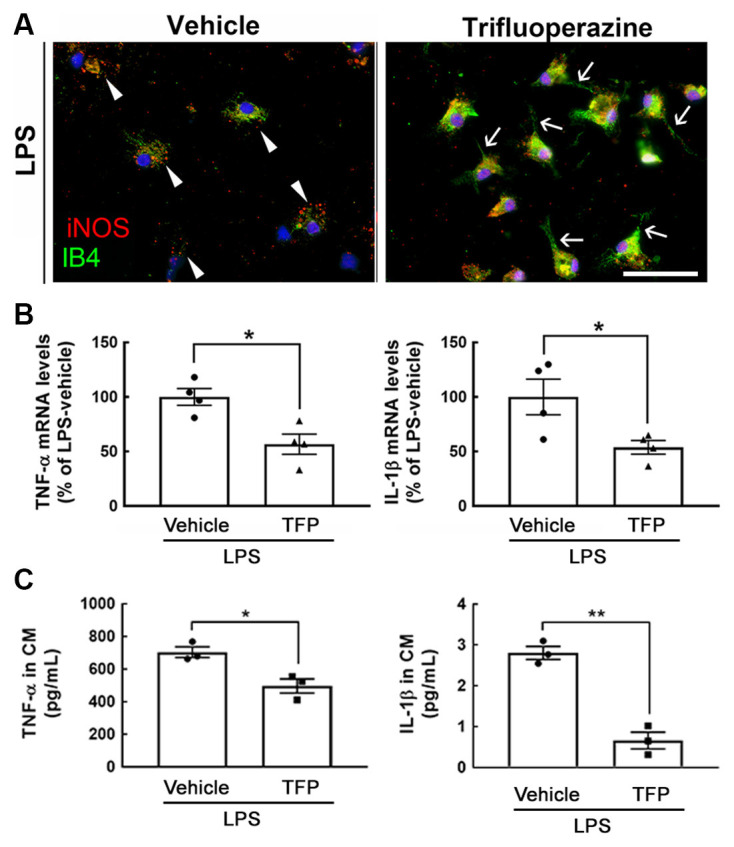
Exposure to TFP suppresses the expression of proinflammatory mediators in glial cultures. **(A)** Mouse microglia were treated with 10 ng/ml of LPS and 1 μM of TFP for 3 h, and then subjected to immunostaining for iNOS (red) and IB4 staining (green) as described in “Materials and Methods” section. Arrowheads indicate iNOS expression significantly in amoeboid microglia, and arrows show microglia with extending cell processes. Scale bar in **(A)** = 50 μm. **(B)** Rat microglia were treated with vehicle or TFP in the presence of LPS for 6 h, and then their TNF-α and IL-1β mRNA expression was measured by QPCR as described in “Materials and Methods” section. **(C)** Mouse hypothalamic mixed glial cells were treated with 1 μM TFP in the presence of LPS (10 ng/ml) for 3 h. The levels of TNF-α and IL-1β in the culture medium were measured using ELISA kits. The data are presented in (**B**; *n* = 4 experiments) and (**C**; *n* = 3 experiments) as the mean ± SEM. *p < 0.05, **p < 0.01 vs. Vehicle.

An *in vitro* study using primary microglia prepared from mouse pups at PD 1–2 was first conducted. As shown in Figure 1A, exposure to TFP at 1 μM for 3 h reduced iNOS immunoreactivity in IB4^+^-microglia under 10 ng/ml of LPS stimulation. We found that iNOS^+^-microglia in the culture only treated with LPS displayed an amoeboid shape (Figure 1A, arrowheads), whereas microglia co-treated with LPS and TFP had a strong IB4 staining, and exhibited extending cell processes (Figure 1A, arrows). This morphological alteration was also observed by CD11b immunofluorescent staining (Supplementary Figure 2A). Note that iNOS expression was undetectable in microglia without LPS stimulation (Supplementary Figure 2B). Alternatively, IB4 fluorescent intensity in TFP-treated microglia showed an increased trend (Supplementary Figure 2C). Moreover, 1 μM of TFP induced the downregulation of proinflammatory cytokines, TNF-α and IL-1β, in LPS-stimulated microglia (Figure 1B). The results from hypothalamic mixed glia stimulated by LPS also indicated that TFP significantly inhibited LPS-induced release of the proinflammatory cytokines (i.e., TNF-α and IL-1β) in the cultures (Figure 1C). Accordingly, the anti-inflammatory ability of TFP in microglia and mixed glia was validated.

**Figure 2:**
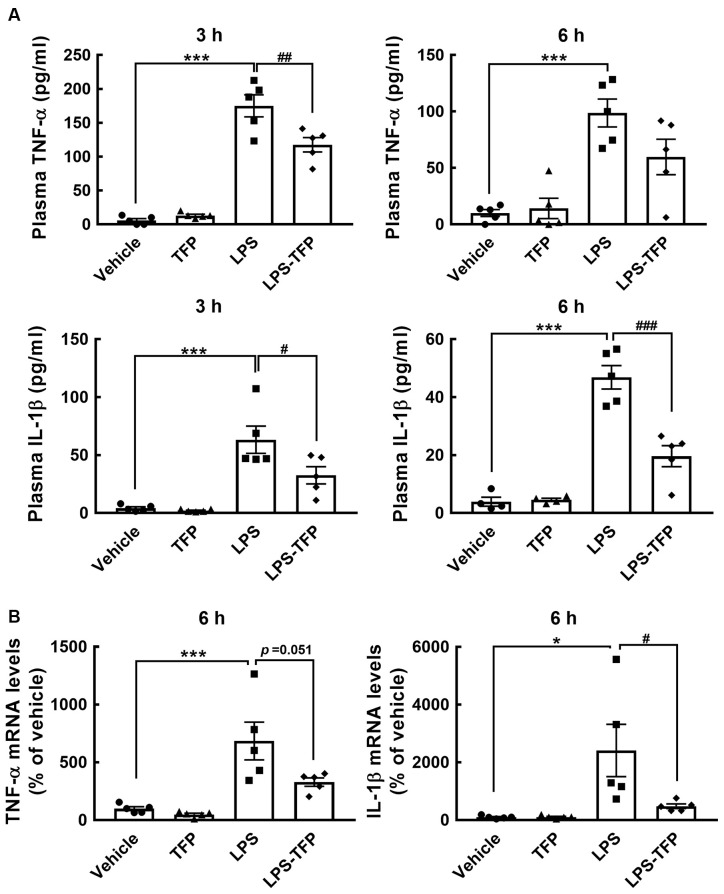
TFP inhibits LPS-induced production of TNF-α and IL-1β in the plasma and hypothalamus. Animals were divided into four groups that received a bolus peripheral injection of vehicle, TFP (2 mg/kg), LPS (1 mg/kg), or LPS plus TFP. Blood samples from the four animal groups were collected at 3 and 6 h post injection for TNF-α and IL-1β ELISAs **(A)**, and then hypothalamic RNA isolation was conducted for the determination of TNF-α and IL-1β mRNA expression **(B)** after animals were sacrificed at 6 h post injection. The data are presented as the mean ± SEM (*n* = 5 animals in each group). Samples from five animals per group were used for most assays, whereas samples from four animals were used for the IL-1β ELISA at 6 h **(A)**. Data are presented as the mean ± SEM (*n* = 5 animals for each group). Each dot represents one animal. *p < 0.05, ***p < 0.001 vs. Vehicle; ^#^*p* < 0.05, ^##^*p* < 0.01, ^###^*p* < 0.001, vs. LPS.

**Figure 3:**
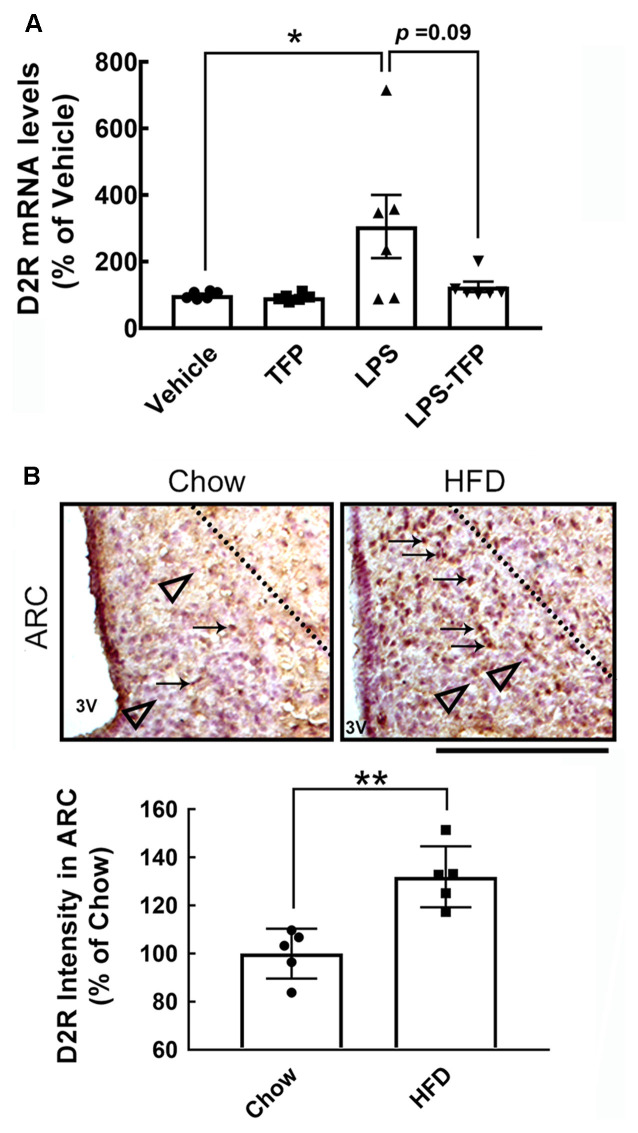
Increased expression of D2R in the hypothalamus after acute peripheral LPS injection or chronic HFD feeding. **(A)** Mice were injected peripherally with the vehicle, TFP (2 mg/kg), LPS (1 mg/kg), or LPS plus TFP as described in the Materials and Methods. The hypothalamic tissues were removed at 6 h post injection for RNA extraction and QPCR analysis to determine the D2R mRNA expression. Each dot represents one animal. The results are presented as the mean ± SEM (*n* = 4 animals for each group). Although D2R expression in the LPS-treated group was not biostatistically greater than that detected in the LPS-treated group (*P* = 0.09), its expression displayed an upregulated trend. **(B)** Mice were sacrificed at 12 weeks after feeding with chow or HFD. Brain sections were prepared as described in the “Materials and Methods” section and subjected to immunohistochemistry for D2R expression. The arrows indicate D2R^+^ cells (brown) in the ARC, which mostly accumulate in HFD-fed mice. The observations were confirmed by quantification of D2R immunoreactive intensity in the ARC. The representative D2R^—^ cells (purple blue) are shown by arrowheads below the dashed line. Each dot represents one immunostained hypothalamic tissue section. The results are presented in **(A)** and **(B)** as the mean ± SEM (*n* = 5–6 animals for each group). Each dot represents one animal. 3V, 3rd ventricle. *p < 0.05 vs. Vehicle **(A)**, **p < 0.01 vs. Chow **(B)**. Scale bar in **(B)**, 100 μm. HFD, high-fat-diet; D2R, dopamine D2 receptor; ARC, arcuate nucleous.

The peripheral acute challenge with 1 mg/kg of LPS is able to trigger systemic inflammation and hypothalamic microglial activation (Borovikova et al., 2000; Qin et al., 2007; Yang et al., 2013). Accordingly, further experiments involving the co-administration of LPS with TFP (2 mg/kg) in mice *via* the i.p. route were designed to examine proinflammatory cytokines in the plasma and hypothalamus, as well as hypothalamic D2R mRNA expression. The plasma levels of TNF-α and IL-1β proteins were significantly elevated at 3 h and 6 h post LPS injection (Figure 2A). This upregulation of TNF-α and IL-1β at the plasma level was effectively diminished by TFP. In parallel, the results also showed that TFP downregulated LPS-stimulated expression of the two proinflammatory cytokine genes in the hypothalamus at 6 h post injection (Figure 2B). These data reveal that the peripheral application of TFP suppressed peripheral and central inflammation. Notably, acute LPS administration peripherally substantially increased D2R mRNA expression in the hypothalamus at 6 h post injection (Figure 3A). Although the suppression of LPS-induced upregulation of D2R mRNA expression by TFP was not statistically significant, hypothalamic D2R mRNA expression in the LPS-TFP group tended to decrease (Figure 3A).

### Trifluoperazine Decreases Plasma TNF-α in Obese Mice

**Figure 4:**
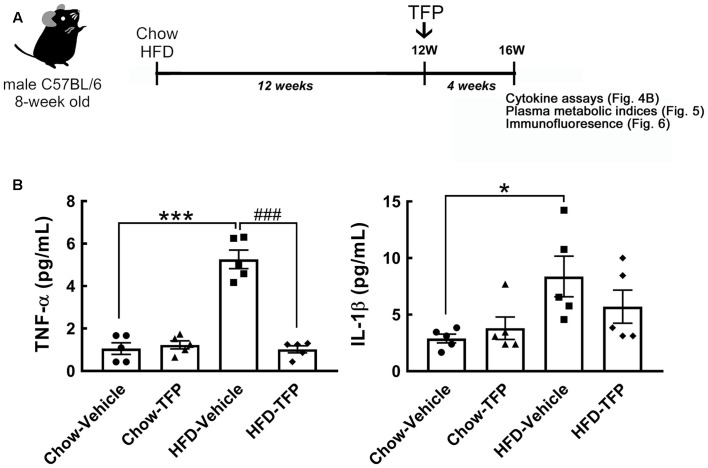
Decreased levels of plasma TNF-α and IL-1β in obese mice treated with TFP for 4 weeks. **(A)** The schematic illustration shows that 8-week-old male C57BL/6 mice were fed chow or HFD for 12 weeks and then injected daily with TFP (2 mg/kg) *via* the i.p. route for another 4 weeks under Chow or HFD feeding (Chow-Vehicle, Chow-TFP, HFD-Vehicle, HFD-TFP). At 16 weeks post feeding, blood samples were collected from animals without fasting for cytokine assays **(B)** and with fasting for the analysis of metabolic indices (Figure 5). The brains were removed for sectioning and immunostaining (Figure 6). **(B)** As indicated above, the plasma samples were subjected to TNF-α or IL-1β ELISA. Data are presented as the mean ± SEM (*n* = 5 animals for each group). Each dot represents one animal. *p < 0.05, ***p < 0.001 vs. Chow-Vehicle; ^###^*p* < 0.001 vs. HFD-Vehicle.

**Figure 5:**
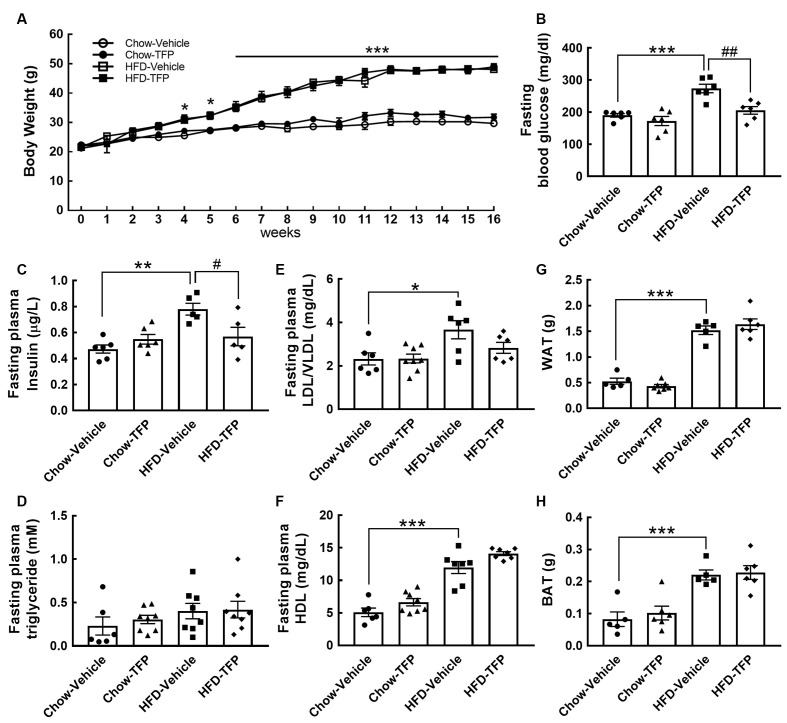
A rise in blood glucose and insulin by chronic HFD feeding was significantly suppressed by treatment with TFP for 4 weeks. **(A)** The body weights of the four animal groups were measured weekly. The body weight gain was significantly greater in HFD-fed animals treated with vehicle or TFP than in chow-fed animals treated with vehicle or TFP. Data are presented as the mean ± SEM (*n* = 6, Chow-Vehicle; *n* = 8, Chow-TFP, HFD-Vehicle, or HFD-TFP). *p < 0.05, ***p < 0.001 vs. chow-vehicle. **(B–H)** At 16 weeks post feeding, the animals were made to fast for 15 h, and then blood samples were collected as described in the “Materials and Methods” section. The samples were subjected to a series of assays including blood glucose **(B)**, insulin **(C)**, triglyceride **(D)**, LDL/VLDL **(E)**, and HDL **(F)**. In addition, WAT **(G)** and BAT **(H)** from the indicated sites in the “Materials and Methods” section were weighed. The results are presented in B-H as the mean ± SEM (*n* = 6–8 animals for each group). Each dot represents one animal. *p < 0.05, **p < 0.01, ***p < 0.001 vs. Chow-Vehicle; ^#^*p* < 0.05, ^##^*p* < 0.01 vs. HFD-Vehicle.

Chronic HFD feeding over 2 months acts as the causal regulator to trigger prolonged microglial activation in the hypothalamic ARC (Huang et al., 2019). An increase in D2R immunoreactivity in the hypothalamic area, especially the ARC, was detected at 12 weeks after HFD feeding when compared to that examined in the chow group (Figure 3B, arrows). This finding, in conjunction with the results shown in Figure 3A, demonstrates that peripheral inflammation either induced by the LPS challenge or chronic HFD feeding can trigger D2R expression in the hypothalamus. Accordingly, daily administration of TFP in mice through the i.p. route started at 12 weeks post HFD feeding and continued for 4 weeks (Figure 4A). The plasma levels of TNF-α and IL-1β were measured at the end of the experiments. As shown in Figure 4B, chronic HFD feeding increased plasma TNF-α and IL-1β. Plasma TNF-α levels declined efficiently after TFP treatment for 4 weeks, although TFP decreased the amount of plasma IL-1β to a lesser extent. Since the body weight of obese mice was not changed by administration of TFP (Figure 5A), the metabolic indices at the end of the experimental time points were examined. We noticed that the levels of blood glucose and plasma insulin were lower in HFD-fed mice receiving treatment with TFP than those detected in HFD-fed animals treated with Vehicle (Figures 5B,C). However, TFP application did not influence the WAT and BAT of obese mice or the plasma levels of LDL/VLDL, triglycerides, or HDL in obese mice (Figures 5B–F). Nevertheless, the findings point to the suppressive effect of TFP on HFD-induced peripheral inflammation.

### Trifluoperazine Suppresses HFD-Induced Gliosis in the Hypothalamus

**Figure 6:**
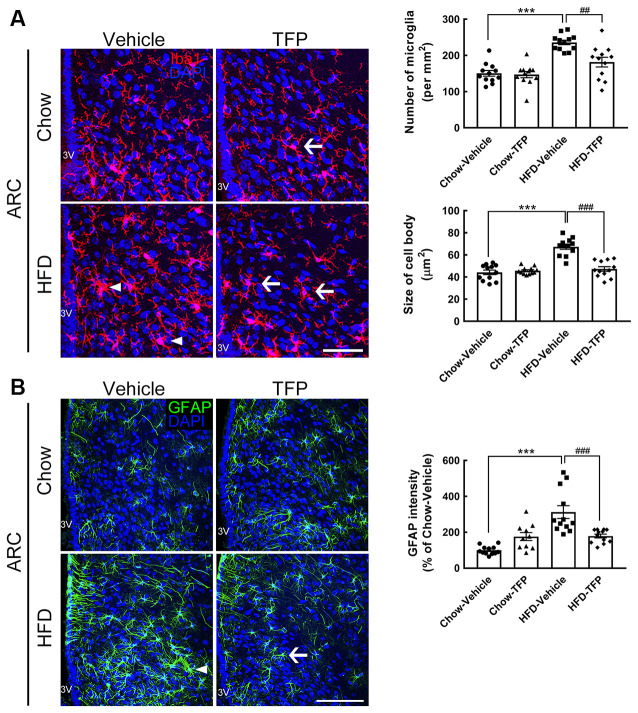
Microglial activation and astrocytic hypertrophy in the ARC of obese mice were attenuated by TFP treatment for 4 weeks. Brain tissue sections were prepared from the four animal groups at 16 weeks post feeding (Figure 4A) and then subjected to Iba1 immunofluorescence (red in **A**) and GFAP immunofluorescence (green in **B**). DAPI nuclear counterstaining (blue in **A,B**) was conducted. Representative active microglia **(A)** and hypertrophic astrocytes **(B)** are indicated by arrowheads in the HFD-vehicle group. Arrows show microglia or astrocytes with a small size and fine processes in chow-TFP or HFD-TFP. The number of Iba1^+^ microglia in the ARC (per mm^2^) and their averaged cell size (μm^2^) in the four animal groups were measured. In addition, the average cell body size of microglia in the two regions was measured. In addition, the intensity of GFAP immunoreactivity in the ARC was quantified. The results are presented as the mean ± SEM (*n* = 12 tissue sections for Iba1 immunostaining from three animals per group; *n* = 9 tissue sections for GFAP immunostaining from three animals per group). Each dot represents one image. 3V, 3rd ventricle. ***p < 0.001 vs. Chow-Vehicle; ^##^*p* < 0.01, ^###^*p* < 0.001 vs. HFD-Vehicle. Scale bar, 50 μm in **(A)**; 100 μm in **(B)**.

Activated microglia and astrocytes in the ARC are the key player to induce hypothalamic inflammation at the acute stage of over-nutrition feeding or chronic HFD feeding (Thaler et al., 2012; Cai and Khor, 2019; Lee et al., 2020). Moreover, astrocytic and microglial activation is characterized by cell morphological changes including cellular process hypertrophy with GFAP upregulation, and an increase in cell numbers and cell body size, respectively (Robel et al., 2011). Thus, to evaluate if daily peripheral injection with TFP attenuated HFD-induced hypothalamic inflammation, we examined the alteration of microglial morphology and astrocytic hypertrophy in the hypothalamic ARC at 16 weeks post HFD feeding and TFP treatment (Figure 4A). Similar to the results shown in our previous study (Huang et al., 2019), activated microglia with a larger cell size were observed in obese mice fed an HFD for 16 weeks when compared to those in the chow group (Figure 6A, arrowheads); moreover, the activated microglia in the ARC of HFD-fed mice increased in number. However, 4 weeks of treatment with TFP significantly reduced the number of ARC microglia and their cell bodies (Figure 6A, arrows). Chronic HFD feeding for 16 weeks not only caused astrocytic hypertrophy but also increased the intensity of astrocytes in the ARC compared to those observed in the chow-fed group (Figure 6B, arrows). HFD-induced astrocytic activation was decreased after daily treatment with TFP for 4 weeks. Note that TFP application for 4 weeks caused insignificant alterations in microglia and astrocytes in the ARC of chow-fed mice. Altogether, daily treatment with TFP for 4 weeks is effective in suppressing HFD-induced peripheral inflammation and ARC gliosis in obese mice.

## Discussion

The findings from others have indicated that the inhibition of hypothalamic inflammation improves the control of glucose homeostasis (Milanski et al., 2009; Posey et al., 2009; Miller and Spencer, 2014), pointing to the crucial role of hypothalamic inflammation in obesity-associated pathogenesis. Here, we show the anti-inflammatory action of TFP on peripheral and hypothalamic gliosis in obese mice. Although TFP administration did not reduce the body weight of the obese mice, the levels of blood glucose and insulin in HFD-fed animals can be suppressed by TFP daily given up to 4 weeks. Yet, despite that TFP action on restoring obesity-associated glucose tolerance or insulin resistance has to be further determined, the study is first to show TFP as a potential anti-inflammatory agent for obesity-associated inflammation.

Hypothalamic inflammation induced by HFD feeding has been linked to the development of obesity (Thaler et al., 2012; Baufeld et al., 2016). Moreover, the selective ablation of microglia in the ARC using the colony-stimulating factor 1 receptor (CSF1R) inhibitor PLX5622 is able to lessen HFD-triggered body weight gain and food intake (Valdearcos et al., 2017). In this study, although microgliosis in the ARC was reduced after the peripheral application of TFP (2 mg/kg) for 1 month, there was no alteration in the body weight of obese mice with or without treatment with TFP. In past studies showing decreased body weight gain by the inhibition of microglial activation (Valdearcos et al., 2017), either CSF1R inhibitor-induced selective ablation of microglia or genetic reduction in microglial inflammatory capacity to restrict hypothalamic inflammation was performed at the beginning of HFD feeding. In addition, the approach used to inhibit microglial proliferation for the prevention of central and peripheral inflammation using intracerebroventricular implantation of an Alzet osmotic minipump containing the antimitotic drug arabinofuranosyl cytidine was conducted before HFD feeding (Andre et al., 2017). Thus, perhaps TFP treatment should start at an earlier time point rather than 3 months later since the animals have been through a 3-month course of HFD feeding at the beginning of TFP application. However, diet-induced body weight gain remained when the 4-week paradigm of TFP administration was performed in the beginning at 2 months after HFD feeding (Supplementary Figure 3A). The results shed light on the function of a 4-week treatment with TFP in blood glucose homeostasis but not in lipid metabolism.

Given that the effect of chronic HFD feeding on the upregulation of body weight, WAT, and BAT was not blocked by a 4-week treatment with TFP, we reason that TFP action for 4 weeks at later time intervals with the continuous stimulation of HFD feeding might not change the animal’s eating behavior under HFD consumption conditions. This is supported by the observations that daily administration of TFP in HFD-fed mice did not alter their food intake in comparison with the HFD-vehicle group (Supplementary Figure 3B). Additionally, neuronal injury in the ARC has been observed in rats receiving an HFD for 1 week (Thaler et al., 2012). Decreased cell numbers of proopiomelanocortin (POMC)-producing neurons, a group of ARC neurons that suppress food intake, have been shown in mice after HFD feeding for 8 months (Thaler et al., 2012). Additionally, it has been reported that HFD exposure altered the distribution of POMC cell volumes, although HFD exposure either 2 or 6 months did not affect the total cell number of POMC neurons in the ARC (Lemus et al., 2015) Accordingly, chronic stimulation of HFD feeding in our study might perturb neural circuits that mediate energy expenditure and the food reward system. Thus, anti-peripheral inflammation or anti-gliosis by a 4-week program of TFP treatment in obese mice receiving long-term HFD feeding is not satisfactory to promote lipid metabolism and mitigate body weight gain.

Dopamine (DA) is well known to mediate immune responses in the periphery through its receptors expressed in immune cells (Arreola et al., 2016). High levels of brain DA and altered amounts of other neurotransmitters have been detected in obese animals compared to those in the lean group along with an increase in inflammatory mediators and oxidative markers (Labban et al., 2020). TFP has been reported to inhibit cytokine release from LPS-stimulated macrophages and dendritic cells and acts as a potential anti-sepsis drug (Park et al., 2019). This is consistent with our observations showing the anti-inflammatory effect of TFP on LPS-stimulated hypothalamic mixed glia and HFD-triggered peripheral inflammation. Moreover, endogenous expression of D2R was found in activated microglia in the infarct site after cerebral ischemia, in conjunction with an *in vitro* study, pointing to DA function in activated microglia-associated neuroinflammation (Huck et al., 2015). The findings have shown that HFD feeding for 4 weeks did not affect hypothalamic D2R gene expression (De Leeuw Van Weenen et al., 2009). Yet our observation in this study indicated that chronic feeding of an HFD for 3 months increased D2R expression in the hypothalamic ARC. This raises the possibility that the inhibitory effect of TFP on the activation of microglia and astrocytes in the ARC of obese mice might occur through D2R action in peripheral immune cells and/or hypothalamic glial cells. However, TFP has been found to act as an inhibitor of a calcium-binding protein calmodulin (CaM) to reduce stroke-induced brain edema (Sylvain et al., 2021), and decrease the release of proinflammatory cytokines in the animal models of LPS-induced endotoxemia and sepsis (Park et al., 2019). Moreover, it has been proposed that TFP action on the inhibition of CaM was derived from its ability in binding to CaM (Colomer et al., 2010). Thus, it still remains to be verified if the reduction of plasma cytokines and ARC gliosis was due to TFP-triggered D2R action on peripheral tissue cells and ARC glial cells.

It has been reported that the inhibition of hypothalamic inflammation leads to a reduction in obesity-induced fasting hyperglycemia derived from abnormal gluconeogenesis and insulin resistance in the liver (Milanski et al., 2012). This study shows that lower levels of blood glucose and insulin were observed in the HFD-fed group with TFP treatment than those detected in HFD-fed animals receiving Vehicle, suggesting that TFP and TFP-like agents may hold potential for the treatment of obesity-associated hyperglycemia. Considering the limited evidence it is difficult to conclude the regulatory role of reduced ARC gliosis by TFP administration in glucose homeostasis under the condition of HFD feeding, the interrelationship of peripheral and hypothalamic inflammation with hyperglycemia remains to be determined by more in-depth investigations. Nevertheless, the study presented here provides constructive information indicating *in vitro* LPS-treated microglia and HFD-fed obese mice as the experimental platform to compare the effect of TFP with that of the other potential D2R drugs not only on anti-obesity-associated peripheral and hypothalamic inflammation but also on anti-obesity-induced metabolic dysfunction.

## Data Availability Statement

The raw data supporting the conclusions of this article will be made available by the authors, without undue reservation.

## Ethics Statement

The animal study was reviewed and approved by the National Cheng Kung University Institutional Animal Care and Use Committee, Tainan, Taiwan.

## Author Contributions

H-TH designed the experiment, conducted the data analysis, and wrote the manuscript. S-FT, the senior author, provided oversight, experimental design, result interpretation, and manuscript preparation/editing. P-CC, P-SC, and Y-MK participated in the discussion, experimental design, and materials. W-TC assisted with imaging analysis. All authors contributed to the article and approved the submitted version.

## Conflict of Interest

The authors declare that the research was conducted in the absence of any commercial or financial relationships that could be construed as a potential conflict of interest.

## Publisher’s Note

All claims expressed in this article are solely those of the authors and do not necessarily represent those of their affiliated organizations, or those of the publisher, the editors and the reviewers. Any product that may be evaluated in this article, or claim that may be made by its manufacturer, is not guaranteed or endorsed by the publisher.

## Footnotes

^1^ http://www.ncku.edu.tw/

^2^ https://www.nc3rs.org.uk/arrive-guidelines

## Abbreviations

ARC: arcuate nucleus
BAT: brown adipose tissue
CaM: calmodulin
CNS: central nervous system
CSF1R: colony-stimulating factor 1 receptor
CyPA: cyclophilin A
DA: dopamine
D2R: dopamine D2 receptor
ELISA: enzyme-linked immunosorbent assay
GFAP: glial fibrillary acidic protein
HDL: high-density lipoprotein
HFD: high-fat diet
LDL/VLDL: low-density lipoprotein/very-low-density lipoprotein
IB4: isolectin B4
IL-1β: interleukin-1β
IL-6: interleukin-6
iNOS: inducible nitric oxide synthase
i.p.: intraperitoneal
LPS: lipopolysaccharide
NIH: National Institutes of Health
QPCR: quantitative real-time polymerase chain reaction
POMC: proopiomelanocortin
TFP: trifluoperazine
TG: triglyceride
TNF-α: tumor necrosis factor-α
WAT: white adipose tissue.

## References

[B1] Andre C. Guzman-Quevedo O. Rey C. Remus-Borel J. Clark S. Castellanos-Jankiewicz A.. (2017). Inhibiting microglia expansion prevents diet-induced hypothalamic and peripheral inflammation. Diabetes 66, 908–919. 10.2337/db16-0586 27903745

[B2] Arreola R. Alvarez-Herrera S. Perez-Sanchez G. Becerril-Villanueva E. Cruz-Fuentes C. Flores-Gutierrez E.O.. (2016). Immunomodulatory effects mediated by dopamine. J. Immunol. Res. 2016:3160486. 10.1155/2016/3160486 27795960 PMC5067323

[B3] Baufeld C. Osterloh A. Prokop S. Miller K. R. Heppner F. L. (2016). High-fat diet-induced brain region-specific phenotypic spectrum of CNS resident microglia. Acta Neuropathol. 132, 361–375. 10.1007/s00401-016-1595-4 27393312 PMC4992033

[B4] Borovikova L. V. Ivanova S. Zhang M. Yang H. Botchkina G. I. Watkins L. R.. (2000). Vagus nerve stimulation attenuates the systemic inflammatory response to endotoxin. Nature 405, 458–462. 10.1038/35013070 10839541

[B5] Cai D. Khor S. (2019). “Hypothalamic microinflammation” paradigm in aging and metabolic diseases. Cell Metab. 30, 19–35. 10.1016/j.cmet.2019.05.021 31269425

[B6] Chowen J. A. Argente J. Horvath T. L. (2013). Uncovering novel roles of nonneuronal cells in body weight homeostasis and obesity. Endocrinology 154, 3001–3007. 10.1210/en.2013-1303 23798599 PMC3749483

[B7] Colomer J. Schmitt A. A. Toone E. J. Means A. R. (2010). Identification and inhibitory properties of a novel Ca(2+)/calmodulin antagonist. Biochemistry 49, 4244–4254. 10.1021/bi1001213 20392081 PMC2868109

[B8] De Leeuw Van Weenen J. E. Hu L. Jansen-Van Zelm K. De Vries M. G. Tamsma J. T. Romijn J. A.. (2009). Four weeks high fat feeding induces insulin resistance without affecting dopamine release or gene expression patterns in the hypothalamus of C57Bl6 mice. Brain Res 1250, 141–148. 10.1016/j.brainres.2008.11.004 19028458

[B9] Guillemot-Legris O. Muccioli G. G. (2017). Obesity-induced neuroinflammation: beyond the hypothalamus. Trends Neurosci. 40, 237–253. 10.1016/j.tins.2017.02.005 28318543

[B10] Hotamisligil G. S. (2006). Inflammation and metabolic disorders. Nature 444, 860–867. 10.1038/nature05485 17167474

[B11] Huang H. T. Chen P. S. Kuo Y. M. Tzeng S. F. (2020). Intermittent peripheral exposure to lipopolysaccharide induces exploratory behavior in mice and regulates brain glial activity in obese mice. J. Neuroinflammation 17:163. 10.1186/s12974-020-01837-x 32450884 PMC7249324

[B12] Huang H. T. Tsai S. F. Wu H. T. Huang H. Y. Hsieh H. H. Kuo Y. M.. (2019). Chronic exposure to high fat diet triggers myelin disruption and interleukin-33 upregulation in hypothalamus. BMC Neurosci. 20:33. 10.1186/s12868-019-0516-6 31291887 PMC6617565

[B13] Huck J. H. Freyer D. Bottcher C. Mladinov M. Muselmann-Genschow C. Thielke M.. (2015). De novo expression of dopamine D2 receptors on microglia after stroke. J. Cereb. Blood Flow. Metab. 35, 1804–1811. 10.1038/jcbfm.2015.128 26104289 PMC4635235

[B14] Jastroch M. Morin S. Tschop M. H. Yi C. X. (2014). The hypothalamic neural-glial network and the metabolic syndrome. Best Pract. Res. Clin. Endocrinol. Metab. 28, 661–671. 10.1016/j.beem.2014.02.002 25256762

[B15] Kanneganti T. D. Dixit V. D. (2012). Immunological complications of obesity. Nat. Immunol. 13, 707–712. 10.1038/ni.2343 22814340

[B16] Konner A. C. Klockener T. Bruning J. C. (2009). Control of energy homeostasis by insulin and leptin: targeting the arcuate nucleus and beyond. Physiol. Behav. 97, 632–638. 10.1016/j.physbeh.2009.03.027 19351541

[B17] Labban R. S. M. Alfawaz H. Almnaizel A. T. Hassan W. M. Bhat R. S. Moubayed N. M.. (2020). High-fat diet-induced obesity and impairment of brain neurotransmitter pool. Transl. Neurosci. 11, 147–160. 10.1515/tnsci-2020-0099 33312720 PMC7705990

[B18] Lee C. H. Suk K. Yu R. Kim M. S. (2020). Cellular contributors to hypothalamic inflammation in obesity. Mol. Cells 43, 431–437. 10.14348/molcells.2020.0055 32392909 PMC7264480

[B19] Lemus M. B. Bayliss J. A. Lockie S. H. Santos V. V. Reichenbach A. Stark R.. (2015). A stereological analysis of NPY, POMC, Orexin, GFAP astrocyte and Iba1 microglia cell number and volume in diet-induced obese male mice. Endocrinology 156, 1701–1713. 10.1210/en.2014-1961 25742051

[B20] Marques L. O. Lima M. S. Soares B. G. (2004). Trifluoperazine for schizophrenia. Cochrane Database Syst. Rev. 2004:CD003545. 10.1002/14651858.CD003545.pub2 14974020 PMC7003674

[B21] Milanski M. Arruda A. P. Coope A. Ignacio-Souza L. M. Nunez C. E. Roman E. A.. (2012). Inhibition of hypothalamic inflammation reverses diet-induced insulin resistance in the liver. Diabetes 61, 1455–1462. 10.2337/db11-0390 22522614 PMC3357298

[B22] Milanski M. Degasperi G. Coope A. Morari J. Denis R. Cintra D. E.. (2009). Saturated fatty acids produce an inflammatory response predominantly through the activation of TLR4 signaling in hypothalamus: implications for the pathogenesis of obesity. J. Neurosci. 29, 359–370. 10.1523/JNEUROSCI.2760-08.2009 19144836 PMC6664935

[B23] Miller A. A. Spencer S. J. (2014). Obesity and neuroinflammation: a pathway to cognitive impairment. Brain Behav. Immun. 42, 10–21. 10.1016/j.bbi.2014.04.001 24727365

[B24] Park J. H. Park H. J. Lee S. E. Kim Y. S. Jang G. Y. Han H. D.. (2019). Repositioning of the antipsychotic drug TFP for sepsis treatment. J. Mol. Med. (Berl) 97, 647–658. 10.1007/s00109-019-01762-4 30848296 PMC6488556

[B25] Pimentel G. D. Ganeshan K. Carvalheira J. B. (2014). Hypothalamic inflammation and the central nervous system control of energy homeostasis. Mol. Cell Endocrinol. 397, 15–22. 10.1016/j.mce.2014.06.005 24952114

[B26] Posey K. A. Clegg D. J. Printz R. L. Byun J. Morton G. J. Vivekanandan-Giri A.. (2009). Hypothalamic proinflammatory lipid accumulation, inflammation and insulin resistance in rats fed a high-fat diet. Am J. Physiol. Endocrinol. Metab. 296, E1003–1012. 10.1152/ajpendo.90377.2008 19116375 PMC2681305

[B27] Purkayastha S. Cai D. (2013). Neuroinflammatory basis of metabolic syndrome. Mol. Metab. 2, 356–363. 10.1016/j.molmet.2013.09.005 24327952 PMC3854982

[B28] Qin L. Wu X. Block M. L. Liu Y. Breese G. R. Hong J. S.. (2007). Systemic LPS causes chronic neuroinflammation and progressive neurodegeneration. Glia 55, 453–462. 10.1002/glia.20467 17203472 PMC2871685

[B29] Robel S. Berninger B. Gotz M. (2011). The stem cell potential of glia: lessons from reactive gliosis. Nat. Rev. Neurosci. 12, 88–104. 10.1038/nrn2978 21248788

[B30] Roudebush R. E. Berry P. L. Layman N. K. Butler L. D. Bryant H. U. (1991). Dissociation of immunosuppression by chlorpromazine and trifluoperazine from pharmacologic activities as dopamine antagonists. Int. J. Immunopharmacol. 13, 961–968. 10.1016/0192-0561(91)90049-d 1684786

[B31] Rueden C. T. Schindelin J. Hiner M. C. Dezonia B. E. Walter A. E. Arena E. T.. (2017). ImageJ2: imageJ for the next generation of scientific image data. BMC Bioinformatics 18:529. 10.1186/s12859-017-1934-z 29187165 PMC5708080

[B32] Sohn J. W. Elmquist J. K. Williams K. W. (2013). Neuronal circuits that regulate feeding behavior and metabolism. Trends Neurosci. 36, 504–512. 10.1016/j.tins.2013.05.003 23790727 PMC3769497

[B33] Sylvain N. J. Salman M. M. Pushie M. J. Hou H. Meher V. Herlo R.. (2021). The effects of trifluoperazine on brain edema, aquaporin-4 expression and metabolic markers during the acute phase of stroke using photothrombotic mouse model. Biochim. Biophys. Acta Biomembr. 1863:183573. 10.1016/j.bbamem.2021.183573 33561476

[B34] Thaler J. P. Yi C. X. Schur E. A. Guyenet S. J. Hwang B. H. Dietrich M. O.. (2012). Obesity is associated with hypothalamic injury in rodents and humans. J. Clin. Invest. 122, 153–162. 10.1172/JCI59660 22201683 PMC3248304

[B35] Tsai S. F. Wu H. T. Chen P. C. Chen Y. W. Yu M. Tzeng S. F.. (2018). Stress aggravates high-fat-diet-induced insulin resistance *via* a mechanism that involves the amygdala and is associated with changes in neuroplasticity. Neuroendocrinology 107, 147–157. 10.1159/000491018 29920496

[B36] Valdearcos M. Douglass J. D. Robblee M. M. Dorfman M. D. Stifler D. R. Bennett M. L.. (2017). Microglial inflammatory signaling orchestrates the hypothalamic immune response to dietary excess and mediates obesity susceptibility. Cell Metab. 26, 185–197.e183. 10.1016/j.cmet.2017.05.015 28683286 PMC5569901

[B37] Valdearcos M. Robblee M. M. Benjamin D. I. Nomura D. K. Xu A. W. Koliwad S. K. (2014). Microglia dictate the impact of saturated fat consumption on hypothalamic inflammation and neuronal function. Cell Rep. 9, 2124–2138. 10.1016/j.celrep.2014.11.018 25497089 PMC4617309

[B38] Wang C. Y. Hsieh Y. T. Fang K. M. Yang C. S. Tzeng S. F. (2016). Reduction of CD200 expression in glioma cells enhances microglia activation and tumor growth. J. Neurosci. Res. 94, 1460–1471. 10.1002/jnr.23922 27629530

[B39] Williams L. M. (2012). Hypothalamic dysfunction in obesity. Proc. Nutr. Soc. 71, 521–533. 10.1017/S002966511200078X 22954151

[B40] Xia Y. Jia C. Xue Q. Jiang J. Xie Y. Wang R.. (2019). Antipsychotic drug trifluoperazine suppresses colorectal cancer by inducing G0/G1 arrest and apoptosis. Front. Pharmacol. 10:1029. 10.3389/fphar.2019.01029 31572198 PMC6753363

[B41] Yang T. T. Lin C. Hsu C. T. Wang T. F. Ke F. Y. Kuo Y. M. (2013). Differential distribution and activation of microglia in the brain of male C57BL/6J mice. Brain Struct. Funct. 218, 1051–1060. 10.1007/s00429-012-0446-x 22886465

