# Supplementary material for: Inhibitory Effects of Trifluoperazine on Peripheral Proinflammatory Cytokine Expression and Hypothalamic Microglia Activation in Obese Mice Induced by Chronic Feeding With High-Fat-Diet
Source: Front Cell Neurosci. 2021 Oct 26;15:752771. doi: 10.3389/fncel.2021.752771 (PMC8576196; doi:10.3389/fncel.2021.752771)
Supplement: Supplementary file 1 [file Data_Sheet_1.PDF]

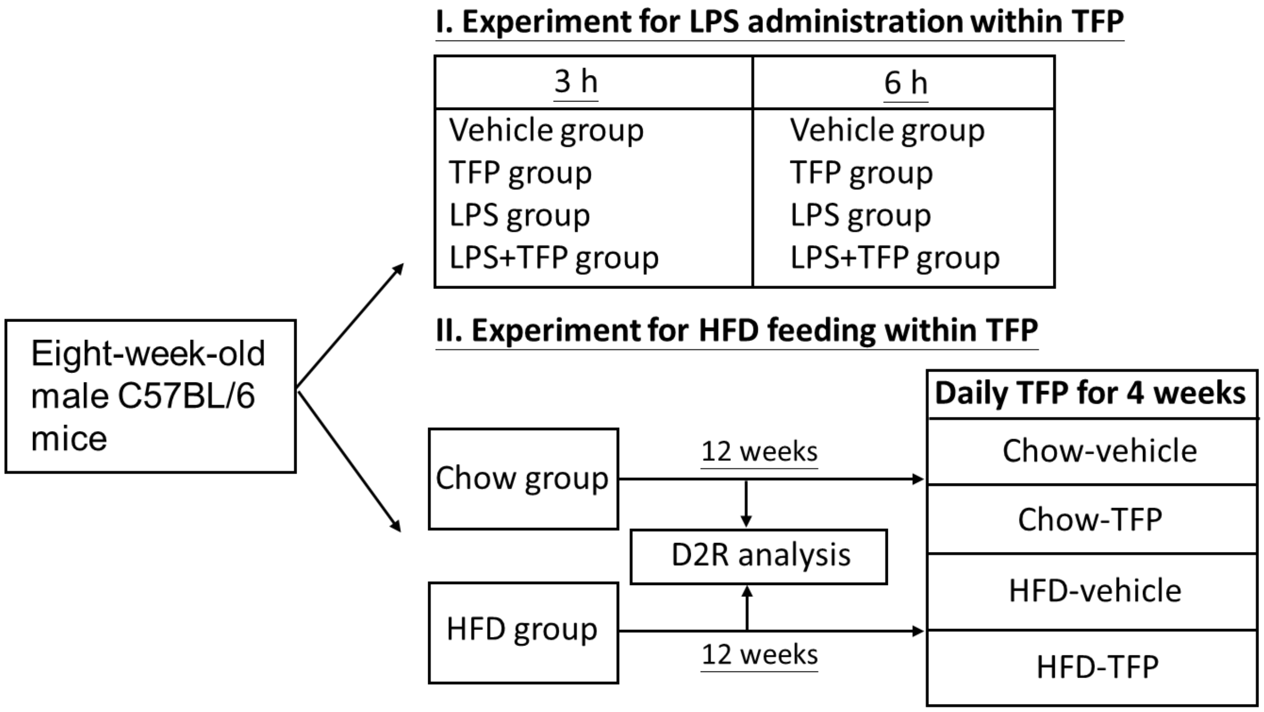

Figure S1. A schematic outline of the animal experimental designs. All the animals were randomly divided into the four groups (Vehicle, TFP, LPS and LPS-TFP) for the acute exposure to LPS (I), and assorted into the four groups (Chow-Vehicle, Chow-TFP, HFD-Vehicle and HFD-TFP) to evaluate the effect of TFP on obese mice (II).

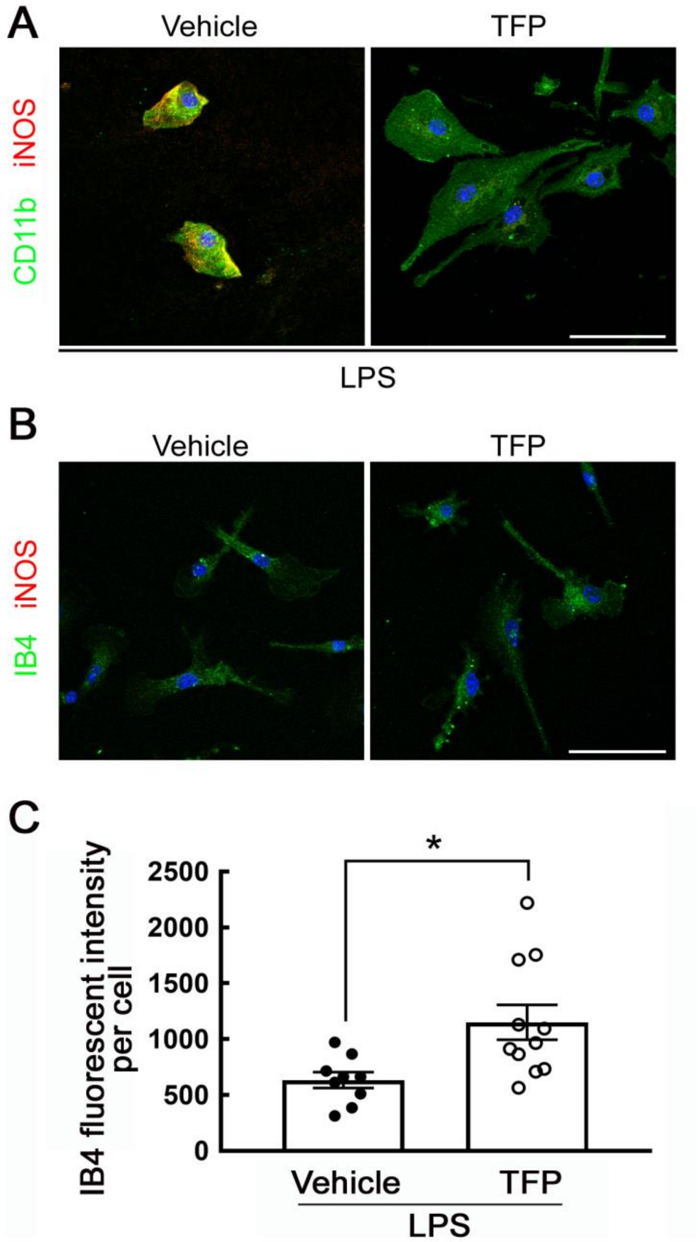

Figure S2. Effect of TFP on microglia in the presence or absence of LPS. (A) Mouse microglia were treated 10 ng/ml of LPS and 1 μM of TFP for 3 h, and then subjected to immunostaining for iNOS (red) and CD11b (green) as described in Materials and Methods. iNOS expression was found significantly in amoeboid microglia (Vehicle), while microglia with extending cell processes was observed in the TFP-treated culture. (B) Mouse microglia were treated with vehicle or TFP in the absence of LPS for 3 h, and then subjected to iNOS immunoflourescence (red) and IB4 staining (green). (C) After treatment of mouse microglia with 1 μM TFP in the presence of LPS (10 ng/ml) for 3 h. The levels of IB4 intensity in cells were quantified using NIH Imaging J. The data are presented as the mean ± SEM from 9 images for Vehicle or from 11 images for TFP. Each dot represents the data from one image that was taken from confocal microscopy using 40X lens. At least 50 cells in the Vehicle or TFP group were measured. *p<0.05 versus Vehicle. Scale bar in A and B, 50 μm.

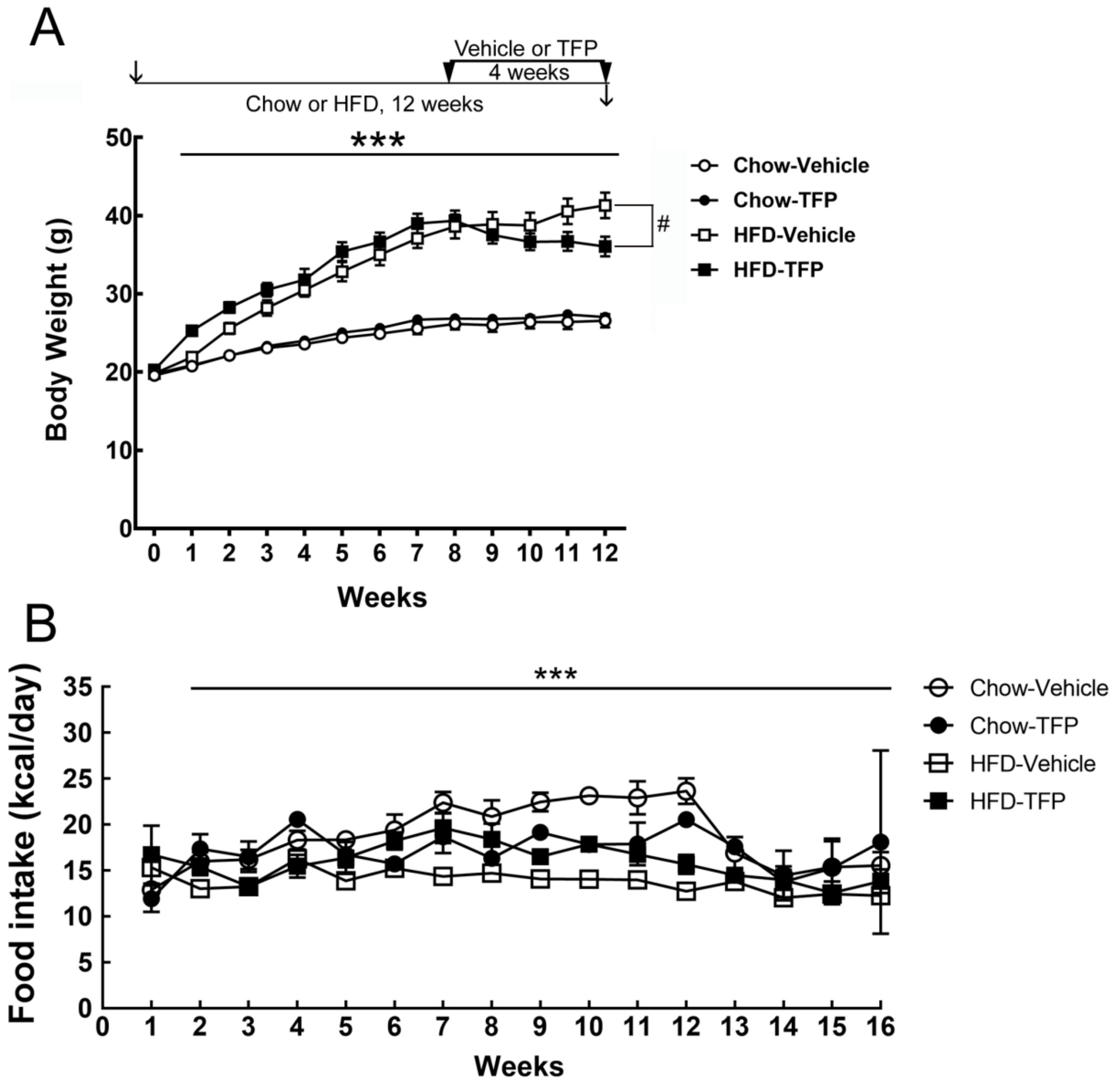

Figure S3. Time-profile examination of the animal body weights and food intake after injections with vehicle or TFP. (A) Animals were divided into the four groups (Chow-Vehicle, HFD-Vehicle, Chow-TFP, HFD-TFP), and then fed by Chow or HFD for 8 weeks. The animal's daily received vehicle and TFP for 4 weeks. The body weights were measured every week. The data are presented as the mean ± SEM (n = 6-8 animals in each group). (B) The food intake of the four animal groups (Chow-Vehicle, HFD-Vehicle, Chow-TFP, HFD-TFP) was measured weekly for 16 weeks. The data are presented as the mean ± SEM (n = 8 animals in each group). #p<0.05 HFD-Vehicle versus HFD-TFP at 12 week; ***p<0.001 in (A) and (B) HFD-Vehicle (or HFD-TFP) versus Chow-Vehicle (or Chow-TFP).
